# *CCND2*, *CTNNB1*, *DDX3X*, *GLI2*, *SMARCA4*, *MYC*, *MYCN*, *PTCH1*, *TP53*, and *MLL2* gene variants and risk of childhood medulloblastoma

**DOI:** 10.1007/s11060-015-1891-1

**Published:** 2015-08-20

**Authors:** Anna M. Dahlin, Mads V. Hollegaard, Carl Wibom, Ulrika Andersson, David M. Hougaard, Isabelle Deltour, Ulf Hjalmars, Beatrice Melin

**Affiliations:** Department of Radiation Sciences, Oncology, Umeå University, Umeå, Sweden; Department of Congenital Disorders, Danish Centre for Neonatal Screening, Statens Serum Institut, Copenhagen, Denmark; Section of Environment and Radiation, International Agency for Research on Cancer, Lyon, France; Unit of Statistics, Bioinformatics and Registry, Danish Cancer Society Research Center, Copenhagen, Denmark

**Keywords:** Medulloblastoma, PNET, Primitive neuroectodermal tumors, Genetic association studies, Genetic variation

## Abstract

Recent studies have described a number of genes that are frequently altered in medulloblastoma tumors and that have putative key roles in the development of the disease. We hypothesized that common germline genetic variations in these genes may be associated with medulloblastoma development. Based on recent publications, we selected 10 genes that were frequently altered in medulloblastoma: *CCND2*, *CTNNB1*, *DDX3X*, *GLI2*, *SMARCA4, MYC*, *MYCN*, *PTCH1*, *TP53*, and *MLL2* (now renamed as *KMT2D*). Common genetic variants (single nucleotide polymorphisms) annotating these genes (*n* = 221) were genotyped in germline DNA (neonatal dried blood spot samples) from 243 childhood medulloblastoma cases and 247 control subjects from Sweden and Denmark. Eight genetic variants annotating three genes in the sonic hedgehog signaling pathway; *CCND2*, *PTCH1*, and *GLI2*, were found to be associated with the risk of medulloblastoma (*P*_combined_ < 0.05). The findings were however not statistically significant following correction for multiple testing by the very stringent Bonferroni method. The results do not support our hypothesis that common germline genetic variants in the ten studied genes are associated with the risk of developing medulloblastoma.

**Electronic supplementary material:**

The online version of this article (doi:10.1007/s11060-015-1891-1) contains supplementary material, which is available to authorized users.

## Introduction

Medulloblastoma is a rare, embryonal tumor of the cerebellum that occurs predominantly in children [1]. Intensive treatment, including surgery, chemotherapy, and radiotherapy, cures about two-thirds of all children that are diagnosed with the disease [1, 2]. However, the children who survive often suffer from serious and disabling late side effects, such as neurocognitive dysfunction and risk of secondary malignancies.

A small subset of medulloblastomas is seen in individuals with predisposing genetic syndromes, caused by germline mutations in *PTCH1* or *SUFU* (Gorlin syndrome), *APC* (Turcot syndrome), and *TP53* (Li Fraumeni syndrome) [3, 4]. Because the tumor occurs early in life, even in very young children and infants, and because there are no established environmental risk factors for medulloblastoma [2], the presence of additional genetic factors that increase the risk of this disease is plausible.

Recent studies have reported a number of genes in which somatic mutations and copy number alterations have been detected in multiple medulloblastoma tumors. Among the most frequently altered genes are well known oncogenes and tumor suppressors (e.g., *MYC*, *TP53*, and *PTCH1*) as well as novel medulloblastoma candidate genes (e.g., *MML2*) [5–10]. The aim of this study was to investigate if common germline genetic variations [single nucleotide polymorphisms (SNPs)] are associated with increased risk of developing medulloblastoma in children and young adults. For this purpose, we applied a target gene approach, focusing on ten of the most frequently altered genes in medulloblastoma tumors.

## Methods

### Study population

In Sweden, 136 medulloblastoma cases born between 1975 and 2002, diagnosed before the age of 25, and present in the Swedish phenylketonuria screening registry were included in the study. Equally many control subjects, matched to each case by year of birth, were drawn from the same biobank. For five cases, matching by year of birth was not possible, and controls were matched by year of birth ± 5 years. Matching by gender was not possible due to an overrepresentation of females among consenting individuals. Nine Swedish case and eight Swedish control samples were removed due to technical issues. In Denmark, 128 medulloblastoma cases born between 1982 and 2008, diagnosed before the age of 20, and present in the Danish Newborn Screening Biobank were included in the study. Equally many control subjects, matched to each case by year of birth and gender, were drawn from the same biobank. One Danish case was removed due to technical issues.

### DNA extraction and genotyping

DNA was extracted from dried blood spot samples (DBSS) collected from each subject close to birth, and stored at the Swedish phenylketonuria screening registry and the Danish Newborn Screening Biobank. Two disks were punched from each participant’s DBSS. DNA was extracted using the Extract-N-amp kit (Sigma-Aldrich) as described previously [11–13]. The extracted DNA was whole-genome-amplified using the REPLIg kit (QIAGEN; Danish samples) or the GenomePlex single cell whole genome amplification (WGA) kit (Sigma-Aldrich; Swedish samples) according to the manufactures’ instructions. Both kits have been shown to perform well in the downstream analyses used in this study [11]. Genotyping was performed using HumanOmni2.5-8 BeadChips (Illumina) at Aros, Denmark. WGA specific cluster files were generated and genotypes called using GenomeStudio V2010.3 (Illumina) and GenomeStudio Genotyping Analysis Module 1.8.4. Four subjects (one Swedish case, one Danish case, and two Danish controls) had a call-rate <97 % and were excluded from further analyses, whereas all other samples had a call-rate >97 %, indicating good DNA quality. SNPs were excluded based on the following criteria in either data set: call-rate <95 %, minor allele frequency <1 %, Hardy–Weinberg test *P* < 1 × 10^−6^, and missing genotypes non-randomly distributed between cases and controls.

### Selection of candidate genes and SNPs

Ten genes (*CCND2*, *CTNNB1*, *DDX3X*, *GLI2*, *SMARCA4*, *MYC*, *MYCN*, *PTCH1*, *TP53* and *MLL2*, Supplementary Table 1) were selected for investigation because they were known to have a well-defined biological function and were among the most commonly altered genes in medulloblastoma tumors [5–10]. Two hundred and twenty-one SNPs annotating these genes fulfilled the criteria for inclusion in both datasets (Supplementary Tables 1, 2).

### Statistical analyses

Odds ratios (ORs) and 95 % confidence intervals (95 % CIs) were calculated for the Swedish and Danish datasets separately using unconditional logistic regression. ORs and *P* values for the combined dataset were then estimated using a fixed-effect model meta-analysis. To account for multiple testing, Bonferroni correction was performed. Statistical calculations and data management were done using PLINK (version 1.07, http://pngu.mgh.harvard.edu/purcell/plink/) [14]. We used principal component analysis (EIGENSOFT [15, 16]) to identify and exclude five Swedish (three cases and two controls) and 11 Danish (six cases and five controls) outlier individuals.

## Results

In the final analyses, 123 medulloblastoma cases and 126 control subjects from Sweden and 120 medulloblastoma cases and 121 control subjects from Denmark were included (Table 1). Risk estimates for 221 investigated genetic variants (annotated to *CCND2*, *CTNNB1*, *DDX3X*, *GLI2*, *SMARCA4, MYC*, *MYCN*, *PTCH1*, *TP53*, and *MLL2*) are listed in Supplementary Table 2. Eight variants annotating *CCND2*, *PTCH1*, and *GLI2* were associated with risk of medulloblastoma (for all eight variants, *P*_combined_ < 0.05; Table 2, Supplementary Table 3). These findings were however not statistically significant after Bonferroni correction for multiple comparisons.

**Table 1:** Study subjects

	Swedish cases	Swedish controls	Danish cases	Danish controls
Total (*n*)	123	126	120	121
Sex				
Male (*n*)	77	61	68	67
Female (*n*)	46	65	52	54

**Table 2:** Genetic variations in *GLI2*, *PTCH1* and *CCND2* and association with medulloblastoma risk

SNP [minor/major (ref) allele]^a^	Gene	OR_combined_ (95 % CI)^b^	*P*_combined_^b^
rs13008945 (G/A)	*GLI2*	0.70 (0.50–0.97)	0.033
rs2121992 (A/G)	*GLI2*	0.72 (0.52–0.99)	0.046
rs4848628 (C/A)	*GLI2*	0.68 (0.48–0.98)	0.039
rs11122821 (G/A)	*GLI2*	0.74 (0.57–0.95)	0.018
rs1992900 (A/G)	*GLI2*	1.32 (1.00–1.75)	0.049
rs77224875 (G/A)	*PTCH1*	0.45 (0.25–0.81)	0.008
rs3217805 (G/C)	*CCND2*	0.67 (0.51–0.87)	0.003
rs4372527 (G/A)	*CCND2*	1.38 (1.04–1.82)	0.024

^a^ Only SNPs with *P*_combined_ < 0.05 are listed in this table. ORs and *P* values for all investigated SNPs are found in Supplementary Table 2

^b^ Risk estimates were calculated in the Swedish and the Danish datasets separately (Supplementary Table 3), and then combined using fixed-effect model meta-analysis

## Discussion

The identification of common germline genetic variation that predispose to medulloblastoma development could increase our understanding of the disease and may facilitate the development of targeted therapeutics and measures of prevention. In our study, eight genetic variants, annotating three genes involved in the sonic hedgehog signaling pathway (*CCND2*, *PTCH1*, and *GLI2*), were indicted as associated with medulloblastoma risk. The associations were however not statistically significant following Bonferroni correction for multiple comparisons, which on the other hand may be overly conservative as many of the variants are in linkage disequilibrium with each other.

Recent studies of the somatic landscape of medulloblastoma tumors have described recurrent alterations, including mutations and copy number aberrations, in the ten genes selected for investigation in the present study [5–10]. Previous knowledge of gene function also contributes to the plausible roles of the selected genes in medulloblastoma tumourigenesis. Rare germline mutations in *PTCH1* are known to cause Gorlin syndrome (also called basal cell naevus syndrome) giving carriers a sensitivity to ionizing radiation and an increased risk of jaw cysts, basal cell carcinoma and about 5 % life time risk for developing medulloblastoma [17]. GLI family zinc finger 2 (*GLI2*) encodes a transcription factor that mediates SHH signaling at the distal end of the pathway and *CCND2* is coding for the cyclin D2 protein, which is a central regulator of cell cycling.

A strength of this study was that all included cases were medulloblastoma, whereas many previous studies have analyzed medulloblastoma combined with other childhood brain tumors [18–20]. However, medulloblastoma is a heterogeneous disease which can be divided into different subgroups based on tumor histology (histological subgroups) or patterns of gene expression in the tumor (molecular subgroups) [21]. If a genetic variant is associated with only one of the medulloblastoma subgroups, the result for this variant would be diluted when analyzing all medulloblastoma together, possibly generating a false negative result. We could not stratify the included subjects by histological or molecular subtypes of their tumors due to the limited size of the study and the fact that we did not have access to a full pathology review or tumor tissue for all cases.

This is one of the first population based studies of potential etiologic common germline genetic variants in medulloblastoma. Increased understanding of genetic variation in genes important in medulloblastoma tumourigenesis could feed into clinical and health science perspectives of intervention and protection against gene-environment interactions. Because medulloblastoma is a rare disease, an international consortium is necessary to increase the number of samples and get statistical power sufficient for genome-wide analyses and in depth interrogation of candidate genes that contribute to medulloblastoma etiology.

## Electronic supplementary material

Supplementary material 1. Supplementary Table 1 Genes included in the present study. Supplementary Table 2 OR and p-values for risk of medulloblastoma for all investigated SNPs. Supplementary Table 3 OR and p-values for eight genetic variants (in *GLI2*, *PTCH1* and *CCND2*), calculated in the Swedish and Danish datasets separately. (PDF 636 kb)
