# Supplementary material for: CCND2, CTNNB1, DDX3X, GLI2, SMARCA4, MYC, MYCN, PTCH1, TP53, and MLL2 gene variants and risk of childhood medulloblastoma
Source: J Neurooncol. 2015 Aug 20;125(1):75–8. doi: 10.1007/s11060-015-1891-1 (PMC4592490; doi:10.1007/s11060-015-1891-1)
Supplement: Supplementary file 1 — Supplementary material 1. Supplementary Table 1 Genes included in the present study. Supplementary Table 2 OR and p-values for risk of medulloblastoma for all investigated SNPs. Supplementary Table 3 OR and p-values for eight genetic variants (in GLI2, PTCH1 and CCND2), calculated in the Swedish and Danish datasets separately. (PDF 636 kb) [file 11060_2015_1891_MOESM1_ESM.pdf]

Supplemental Material for

CCND2, *CTNNB1*, *DDX3X*, *GLI2*, *SMARCA4*, *MYC*, *MYCN*, *PTCH1*, *TP53*, and *MLL2* gene variants and risk of childhood medulloblastoma

Anna M Dahlin[1], Mads V Hollegaard[2], Carl Wibom[1], Ulrika Andersson[1], David M Hougaard[2], Isabelle Deltour[3,4], Ulf Hjalmars[1], and Beatrice Melin[1]

[1] Department of Radiation Sciences, Oncology, Umeå University, 901 87 Umeå, Sweden

[2] Danish Centre for Neonatal Screening, Department of Congenital Disorders, Statens Serum Institut, DK-2300, Copenhagen, Denmark

[3] International Agency for Research on Cancer, Section of Environment and Radiation, 69372 Lyon, France

[4] Danish Cancer Society Research Center, Unit of Statistics, Bioinformatics and Registry, DK-2100 Copenhagen, Denmark

Supplementary Table 1. Genes included in the present study

Chr	Gene start[a]	Gene end[a]	Gene	Number of SNPs	
2	16080686	16087129	*MYCN*	1	Recurrently amplified in medulloblastoma tumors [1-3].
2	121493199	121750229	*GLI2*	94	Recurrently amplified in medulloblastoma tumors, particularly in the SHH subgroup [2]. Mediates SHH signaling.
3	41236328	41301587	*CTNNB1*	20	Recurrently mutated in medulloblastoma tumors, particularly in the WNT subgroup [3-6].
8	128747680	128753674	*MYC*	1	Recurrently amplified in medulloblastoma tumors [1-4].
9	98205262	98279339	*PTCH1*	22	Recurrently mutated in medulloblastoma tumors, particularly in the SHH subgroup [3-6].
12	4382938	4414516	*CCND2*	27	Recurrently amplified in medulloblastoma tumors [2]. Involved in cell cycle regulation.
12	49412758	49453557	*MLL2* (*KMT2D*)	9	Recurrently mutated in medulloblastoma tumors [3-6].
17	7565097	7590856	*TP53*	9	Recurrently mutated in medulloblastoma tumors [3-6].
19	11071598	11176071	*SMARCA4*	28	Recurrently mutated in medulloblastoma tumors, particularly in the WNT subgroup [3-6].
X	41192651	41223725	*DDX3X*	10	Recurrently mutated in medulloblastoma tumors, particularly in the WNT subgroup [3, 5, 6].

[a] Ensamble gene annotations (GRCh37.p8) were obtained from MartView (http://biomart.org)

SHH, sonic hedgehog.

Supplementary Table 2. OR and p-values for risk of medulloblastoma for all investigated SNPs

SNP (minor/major(ref) allele)	chr	bp (GRCh37/hg19)	OR_{comb}[a]	P_{comb}[a]	Gene
rs922 (A/G)	2	16086469	1.20	0.236	*MYCN*
rs67611826 (G/A)	2	121500265	1.26	0.084	*GLI2*
rs62150664 (A/G)	2	121501287	0.94	0.681	*GLI2*
rs13008945 (G/A)	2	121506802	0.70	0.033	*GLI2*
rs6757829 (G/A)	2	121509295	0.75	0.244	*GLI2*
rs776501 (A/G)	2	121509378	0.80	0.102	*GLI2*
rs776504 (A/C)	2	121513197	1.28	0.065	*GLI2*
rs11901641 (C/A)	2	121513993	0.93	0.631	*GLI2*
rs13034141 (C/A)	2	121515390	0.72	0.076	*GLI2*
rs707490 (A/G)	2	121517174	1.27	0.071	*GLI2*
rs4848627 (G/A)	2	121530262	0.75	0.078	*GLI2*
rs11677381 (A/G)	2	121530351	1.54	0.086	*GLI2*
rs2121992 (A/G)	2	121532932	0.72	0.046	*GLI2*
rs17389953 (C/A)	2	121532983	0.92	0.578	*GLI2*
rs4848628 (C/A)	2	121536569	0.68	0.039	*GLI2*
rs79503443 (A/G)	2	121537891	0.66	0.374	*GLI2*
rs2053989 (G/A)	2	121544316	1.13	0.380	*GLI2*
rs7605011 (A/G)	2	121551822	1.36	0.218	*GLI2*
rs3820723 (A/C)	2	121552558	1.32	0.258	*GLI2*
rs7569100 (G/A)	2	121554317	0.92	0.754	*GLI2*
rs36124185 (G/A)	2	121558036	0.96	0.763	*GLI2*
rs4848119 (A/G)	2	121559113	1.06	0.638	*GLI2*
rs4848632 (A/G)	2	121562595	1.06	0.649	*GLI2*
rs76201712 (A/G)	2	121563734	1.13	0.517	*GLI2*
rs55633297 (G/A)	2	121565142	1.05	0.845	*GLI2*
rs895485 (G/A)	2	121570665	1.29	0.058	*GLI2*
rs4848636 (A/G)	2	121574575	0.61	0.349	*GLI2*
rs2871627 (A/G)	2	121578394	0.61	0.354	*GLI2*
rs895491 (G/A)	2	121578827	1.25	0.093	*GLI2*
rs11122821 (G/A)	2	121579810	0.74	0.018	*GLI2*
rs11122822 (A/G)	2	121579866	1.21	0.139	*GLI2*
rs7561607 (A/G)	2	121580431	1.23	0.104	*GLI2*
rs116029714 (G/A)	2	121581093	1.24	0.755	*GLI2*
rs2310896 (A/G)	2	121581416	1.08	0.698	*GLI2*
rs58292323 (G/A)	2	121581586	0.65	0.161	*GLI2*
rs4848640 (A/G)	2	121585366	0.74	0.451	*GLI2*
rs1992900 (A/G)	2	121588532	1.32	0.049	*GLI2*
rs895479 (G/A)	2	121589440	1.15	0.443	*GLI2*
rs13393992 (A/G)	2	121591328	1.30	0.144	*GLI2*

rs11122826 (A/C)	2	121591950	1.20	0.474	*GLI2*
rs17005273 (A/G)	2	121592523	0.98	0.920	*GLI2*
rs11885290 (G/A)	2	121592733	0.98	0.876	*GLI2*
rs7559777 (A/C)	2	121595616	1.05	0.718	*GLI2*
rs74641446 (G/A)	2	121595667	0.98	0.943	*GLI2*
rs72971958 (G/A)	2	121598502	1.04	0.846	*GLI2*
rs7557372 (A/C)	2	121598768	0.91	0.575	*GLI2*
rs17005295 (G/A)	2	121600223	1.01	0.941	*GLI2*
rs4848641 (A/G)	2	121603584	0.97	0.830	*GLI2*
rs55917638 (A/G)	2	121606405	0.70	0.470	*GLI2*
rs67124142 (G/A)	2	121607893	1.04	0.743	*GLI2*
rs2166898 (A/G)	2	121612659	0.76	0.097	*GLI2*
rs55706422 (G/A)	2	121613509	1.03	0.807	*GLI2*
rs4848643 (A/G)	2	121618625	1.14	0.334	*GLI2*
rs4143116 (A/G)	2	121620828	1.03	0.839	*GLI2*
rs4848126 (A/G)	2	121627175	1.10	0.462	*GLI2*
rs277554 (G/A)	2	121631384	1.15	0.283	*GLI2*
rs277552 (A/G)	2	121632454	1.16	0.299	*GLI2*
rs55751863 (A/G)	2	121632865	1.02	0.928	*GLI2*
rs62150745 (A/G)	2	121633571	0.84	0.600	*GLI2*
rs277550 (A/G)	2	121634010	1.18	0.222	*GLI2*
rs72835595 (C/A)	2	121640679	1.24	0.625	*GLI2*
rs6541743 (A/G)	2	121647802	1.15	0.292	*GLI2*
rs12478509 (G/A)	2	121648806	0.88	0.527	*GLI2*
rs10199310 (A/G)	2	121649279	0.93	0.706	*GLI2*
rs79465951 (A/G)	2	121651189	1.19	0.409	*GLI2*
rs277536 (A/G)	2	121652747	1.06	0.647	*GLI2*
rs277539 (G/A)	2	121653647	1.05	0.703	*GLI2*
rs776189 (A/G)	2	121655419	1.54	0.096	*GLI2*
rs277541 (A/G)	2	121656429	1.23	0.530	*GLI2*
rs10173670 (C/A)	2	121657322	2.01	0.088	*GLI2*
rs11675340 (A/G)	2	121665196	0.97	0.833	*GLI2*
rs1187934 (A/G)	2	121667962	1.23	0.530	*GLI2*
rs1992902 (G/A)	2	121670602	1.04	0.862	*GLI2*
rs1992901 (A/G)	2	121670669	0.87	0.283	*GLI2*
rs277524 (A/G)	2	121670757	0.83	0.154	*GLI2*
rs10206938 (G/A)	2	121675238	1.03	0.934	*GLI2*
rs895549 (A/G)	2	121676484	1.17	0.582	*GLI2*
rs895547 (A/G)	2	121676655	1.18	0.573	*GLI2*
rs72955383 (A/G)	2	121681721	1.69	0.292	*GLI2*
rs114247324 (G/A)	2	121682216	0.78	0.634	*GLI2*
rs6750250 (G/A)	2	121685681	0.93	0.835	*GLI2*
rs4848648 (G/A)	2	121686439	1.25	0.578	*GLI2*

rs4848649 (A/G)	2	121686533	1.02	0.970	*GLI2*
rs6541745 (A/G)	2	121687129	1.18	0.564	*GLI2*
rs10167579 (A/G)	2	121699964	1.21	0.597	*GLI2*
rs12711535 (G/A)	2	121709536	1.25	0.461	*GLI2*
rs895545 (G/A)	2	121721467	1.21	0.543	*GLI2*
rs13396540 (C/A)	2	121726151	1.03	0.930	*GLI2*
rs280199 (A/G)	2	121733476	0.89	0.419	*GLI2*
rs3768696 (G/A)	2	121733538	0.91	0.495	*GLI2*
rs7588213 (C/A)	2	121735335	0.93	0.618	*GLI2*
rs280194 (G/A)	2	121738820	0.94	0.642	*GLI2*
rs7577521 (A/G)	2	121739875	0.95	0.703	*GLI2*
rs280198 (A/G)	2	121741762	0.92	0.537	*GLI2*
rs116677865 (G/A)	2	121743169	1.16	0.777	*GLI2*
rs11564430 (A/G)	3	41239693	1.13	0.812	*CTNNB1*
rs3915129 (C/A)	3	41243742	0.94	0.612	*CTNNB1*
rs115813431 (C/A)	3	41244376	0.62	0.249	*CTNNB1*
rs12492719 (C/A)	3	41245731	1.06	0.851	*CTNNB1*
rs73081753 (G/A)	3	41245804	1.02	0.964	*CTNNB1*
rs13075993 (A/G)	3	41260017	0.93	0.562	*CTNNB1*
rs11564445 (A/G)	3	41267752	0.76	0.393	*CTNNB1*
rs11564447 (C/A)	3	41268354	0.95	0.892	*CTNNB1*
rs4135385 (G/A)	3	41279440	0.94	0.696	*CTNNB1*
rs2953 (C/A)	3	41281388	0.92	0.530	*CTNNB1*
rs113152028 (G/A)	3	41283882	1.01	0.982	*CTNNB1*
rs9883073 (A/C)	3	41284536	0.93	0.571	*CTNNB1*
rs79668667 (A/G)	3	41284947	1.02	0.975	*CTNNB1*
rs11711946 (G/A)	3	41287018	0.91	0.456	*CTNNB1*
rs6803275 (G/A)	3	41287455	1.06	0.851	*CTNNB1*
rs9850653 (A/G)	3	41292736	0.99	0.977	*CTNNB1*
rs11129896 (G/A)	3	41295767	1.01	0.909	*CTNNB1*
rs9849022 (C/A)	3	41297523	0.99	0.935	*CTNNB1*
rs73081797 (A/G)	3	41299516	1.02	0.892	*CTNNB1*
rs2293300 (C/A)	3	41301534	0.99	0.967	*CTNNB1*
rs10110283 (A/G)	8	128752178	0.75	0.434	*MYC*
rs28485160 (G/A)	9	98205645	0.80	0.332	*PTCH1*
rs16909865 (G/C)	9	98207302	0.78	0.284	*PTCH1*
rs2134721 (A/G)	9	98215306	0.94	0.692	*PTCH1*
rs28457693 (G/A)	9	98217348	0.98	0.917	*PTCH1*
rs2274693 (A/G)	9	98229297	0.96	0.811	*PTCH1*
rs16909898 (G/A)	9	98231008	0.90	0.612	*PTCH1*
rs2066836 (A/G)	9	98238358	0.98	0.882	*PTCH1*
rs2277183 (G/A)	9	98238860	1.54	0.419	*PTCH1*
rs574688 (G/C)	9	98239190	1.08	0.583	*PTCH1*

rs2297086 (A/G)	9	98240120	1.00	0.981	*PTCH1*
rs2297087 (G/A)	9	98242925	0.94	0.700	*PTCH1*
rs17369383 (G/A)	9	98245974	0.94	0.700	*PTCH1*
rs2282041 (G/A)	9	98248387	1.04	0.849	*PTCH1*
rs78400819 (A/G)	9	98248780	0.49	0.113	*PTCH1*
rs77224875 (G/A)	9	98254218	0.45	0.008	*PTCH1*
rs473902 (C/A)	9	98256235	0.87	0.511	*PTCH1*
rs473902 (C/A)	9	98256235	0.87	0.524	*PTCH1*
rs10512248 (C/A)	9	98259703	1.04	0.800	*PTCH1*
rs60417486 (A/G)	9	98262178	0.97	0.904	*PTCH1*
rs16909922 (G/A)	9	98265901	0.97	0.904	*PTCH1*
rs34241213 (A/G)	9	98272006	1.68	0.151	*PTCH1*
rs74499984 (C/A)	9	98272491	1.42	0.465	*PTCH1*
rs3217795 (G/A)	12	4386064	1.12	0.660	*CCND2*
rs3217805 (G/C)	12	4388084	0.67	0.003	*CCND2*
rs4372527 (G/A)	12	4388914	1.38	0.024	*CCND2*
rs11063072 (G/A)	12	4389158	0.87	0.625	*CCND2*
rs3217818 (G/A)	12	4390326	0.99	0.979	*CCND2*
rs3217830 (A/G)	12	4392530	1.29	0.072	*CCND2*
rs3217833 (A/G)	12	4393474	1.10	0.591	*CCND2*
rs3217840 (G/A)	12	4394877	1.29	0.067	*CCND2*
rs3217863 (A/G)	12	4399496	0.66	0.055	*CCND2*
rs3217867 (G/A)	12	4399917	0.91	0.529	*CCND2*
rs3217871 (C/A)	12	4400569	0.66	0.290	*CCND2*
rs3217874 (A/G)	12	4400808	1.05	0.723	*CCND2*
rs3217875 (G/A)	12	4401201	0.81	0.550	*CCND2*
rs3217882 (A/G)	12	4402817	0.97	0.840	*CCND2*
rs3217889 (A/G)	12	4403771	0.91	0.850	*CCND2*
rs3217898 (G/A)	12	4404376	1.23	0.132	*CCND2*
rs3217901 (G/A)	12	4405389	1.04	0.775	*CCND2*
rs3217905 (G/A)	12	4405686	0.93	0.590	*CCND2*
rs3217906 (G/A)	12	4405804	1.00	0.996	*CCND2*
rs10849028 (C/A)	12	4405850	0.82	0.603	*CCND2*
rs12299509 (G/A)	12	4406281	0.89	0.376	*CCND2*
rs3217907 (A/C)	12	4406836	0.78	0.055	*CCND2*
rs3217916 (G/A)	12	4408673	1.16	0.319	*CCND2*
rs3217925 (A/G)	12	4411639	1.07	0.659	*CCND2*
rs3217926 (G/A)	12	4411683	1.22	0.115	*CCND2*
rs1049612 (G/A)	12	4412762	1.23	0.107	*CCND2*
rs3217933 (G/A)	12	4413000	0.77	0.075	*CCND2*
rs7975791 (A/G)	12	49413486	1.02	0.953	*MLL2 (KMT2D)*
rs10875912 (G/A)	12	49416944	1.13	0.380	*MLL2 (KMT2D)*
rs55776396 (C/A)	12	49418435	1.15	0.688	*MLL2 (KMT2D)*

rs117856947 (C/A)	12	49422033	0.66	0.287	*MLL2 (KMT2D)*
rs10875914 (G/A)	12	49422094	1.11	0.421	*MLL2 (KMT2D)*
rs80132640 (G/A)	12	49426460	1.02	0.954	*MLL2 (KMT2D)*
rs10875915 (A/G)	12	49430094	1.15	0.309	*MLL2 (KMT2D)*
rs2304275 (G/A)	12	49442813	1.14	0.372	*MLL2 (KMT2D)*
rs833836 (A/G)	12	49451459	0.96	0.794	*MLL2 (KMT2D)*
rs118051719 (A/G)	17	7566428	1.52	0.460	*TP53*
rs9893249 (G/A)	17	7570189	0.86	0.261	*TP53*
rs9914052 (G/C)	17	7570823	0.81	0.546	*TP53*
rs17881556 (G/A)	17	7570869	0.97	0.926	*TP53*
rs858528 (A/G)	17	7574936	1.37	0.112	*TP53*
rs1625895 (A/G)	17	7578115	1.29	0.204	*TP53*
rs2909430 (G/A)	17	7578645	1.34	0.135	*TP53*
rs11652704 (G/A)	17	7584400	0.87	0.536	*TP53*
rs17881035 (G/A)	17	7587899	0.73	0.262	*TP53*
rs11879293 (A/G)	19	11072610	1.00	0.995	*SMARCA4*
rs2163800 (G/A)	19	11075706	1.04	0.786	*SMARCA4*
rs73011369 (A/G)	19	11077662	1.08	0.773	*SMARCA4*
rs12610374 (A/G)	19	11080921	0.94	0.681	*SMARCA4*
rs4804553 (G/A)	19	11081405	0.94	0.681	*SMARCA4*
rs4804554 (A/C)	19	11083210	0.98	0.910	*SMARCA4*
rs17001070 (G/A)	19	11086746	1.08	0.826	*SMARCA4*
rs12459603 (A/G)	19	11089846	1.36	0.371	*SMARCA4*
rs11672595 (A/G)	19	11094494	0.72	0.382	*SMARCA4*
rs76614166 (G/A)	19	11104243	0.59	0.251	*SMARCA4*
rs17001075 (G/A)	19	11105593	1.13	0.713	*SMARCA4*
rs7935 (G/A)	19	11105608	0.98	0.891	*SMARCA4*
rs57900086 (G/A)	19	11109468	0.97	0.921	*SMARCA4*
rs17001076 (A/G)	19	11113520	0.98	0.933	*SMARCA4*
rs17001078 (G/A)	19	11113651	1.00	0.985	*SMARCA4*
rs12983316 (G/A)	19	11114352	1.05	0.778	*SMARCA4*
rs13345127 (A/G)	19	11116266	0.95	0.788	*SMARCA4*
rs73013166 (G/A)	19	11126160	1.06	0.840	*SMARCA4*
rs73013169 (A/C)	19	11127069	0.98	0.901	*SMARCA4*
rs17001095 (G/A)	19	11133272	1.00	0.995	*SMARCA4*
rs56167249 (G/A)	19	11157423	1.05	0.808	*SMARCA4*
rs77287598 (A/G)	19	11159449	0.69	0.234	*SMARCA4*
rs6511719 (G/A)	19	11164790	1.06	0.838	*SMARCA4*
rs3786724 (G/A)	19	11166791	1.00	0.998	*SMARCA4*
rs3786725 (A/G)	19	11166827	1.00	0.991	*SMARCA4*
rs7258189 (G/A)	19	11169947	0.94	0.646	*SMARCA4*
rs8099996 (G/A)	19	11174625	0.94	0.653	*SMARCA4*
rs73013198 (A/G)	19	11174742	1.01	0.954	*SMARCA4*

rs4358953 (A/G)	X	41196417	0.88	0.374	*DDX3X*
rs11542970 (G/A)	X	41197200	1.00	0.997	*DDX3X*
rs953114 (C/A)	X	41197542	0.87	0.339	*DDX3X*
rs2275943 (G/A)	X	41198456	0.94	0.753	*DDX3X*
rs6610545 (G/A)	X	41198715	0.88	0.379	*DDX3X*
rs12851342 (C/A)	X	41199626	1.02	0.936	*DDX3X*
rs4827268 (A/G)	X	41199812	0.66	0.204	*DDX3X*
rs10521420 (A/G)	X	41202159	0.65	0.198	*DDX3X*
rs72626410 (A/G)	X	41210256	0.66	0.204	*DDX3X*
rs7877664 (A/G)	X	41210942	1.02	0.910	*DDX3X*

[a] Fixed-effect model meta-analysis combining the results from the Swedish and the Danish datasets

Supplementary Table 3. OR and p-values for eight genetic variants (in *GLI2*, *PTCH1* and *CCND2)*, calculated in the Swedish and Danish datasets separately.

SNP (minor/major(ref) allele)	Gene	OR_{Sv}[a] (95% CI)	P_{Sv}[a]	OR_{Dk}[b] (95% CI)	P_{Dk}[b]
rs13008945 (G/A)	*GLI2*	0.57 (0.35-0.93)	0.024	0.83 (0.53-1.30)	0.411
rs2121992 (A/G)	*GLI2*	0.51 (0.31-0.83)	0.007	0.94 (0.61-1.45)	0.778
rs4848628 (C/A)	*GLI2*	0.48 (0.27-0.84)	0.011	0.87 (0.55-1.40)	0.577
rs11122821 (G/A)	*GLI2*	0.68 (0.48-0.97)	0.032	0.80 (0.56-1.15)	0.232
rs1992900 (A/G)	*GLI2*	1.39 (0.93-2.07)	0.104	1.26 (0.85-1.86)	0.247
rs77224875 (G/A)	*PTCH1*	0.37 (0.16-0.87)	0.023	0.54 (0.25-1.19)	0.128
rs3217805 (G/C)	*CCND2*	0.69 (0.47-1.01)	0.058	0.65 (0.45-0.93)	0.018
rs4372527 (G/A)	*CCND2*	1.17 (0.80-1.73)	0.421	1.64 (1.10-2.45)	0.016

[a] OR, 95% CI, and p-value calculated using unconditional logistic regression in the Swedish dataset.
[b] OR, 95% CI, and p-value calculated using unconditional logistic regression in the Danish dataset.

Supplementary References

1. Northcott PA, Nakahara Y, Wu X, et al (2009) Multiple recurrent genetic events converge on control of histone lysine methylation in medulloblastoma. Nat Genet 41: 465-472. doi:10.1038/ng.336

2. Northcott PA, Shih DJ, Peacock J, et al (2012) Subgroup-specific structural variation across 1,000 medulloblastoma genomes. Nature 488: 49-56. doi:10.1038/nature11327

3. Robinson G, Parker M, Kranenburg TA, et al (2012) Novel mutations target distinct subgroups of medulloblastoma. Nature 488: 43-48. doi:10.1038/nature11213

4. Parsons DW, Li M, Zhang X, et al (2011) The genetic landscape of the childhood cancer medulloblastoma. Science 331: 435-439. doi:10.1126/science.1198056

5. Pugh TJ, Weeraratne SD, Archer TC, et al (2012) Medulloblastoma exome sequencing uncovers subtype-specific somatic mutations. Nature 488: 106-110. doi:10.1038/nature11329

6. Jones DT, Jager N, Kool M, et al (2012) Dissecting the genomic complexity underlying medulloblastoma. Nature 488: 100-105. doi:10.1038/nature11284
